# Efficacy of acupoint stimulation as a treatment for uremic pruritus: A systematic review and meta-analysis

**DOI:** 10.3389/fmed.2022.1036072

**Published:** 2022-12-01

**Authors:** Ping-Hsun Lu, Chia-Hsiang Chung, Hui-En Chuo, I-Hsin Lin, Po-Hsuan Lu

**Affiliations:** ^1^Department of Chinese Medicine, Taipei Tzu Chi Hospital, Buddhist Tzu Chi Medical Foundation, New Taipei City, Taiwan; ^2^School of Post-baccalaureate Chinese Medicine, Tzu Chi University, Hualien, Taiwan; ^3^Department of Dermatology, MacKay Memorial Hospital, Taipei, Taiwan; ^4^Department of Medicine, MacKay Medical College, New Taipei City, Taiwan

**Keywords:** acupoint injection, acupressure, acupuncture, chronic kidney disease, uremic pruritus

## Abstract

**Background:**

Uremic pruritus causes sleep disturbances, poor quality of life, and increased morbidity in patients with chronic kidney disease. Acupuncture has been shown to improve uremic pruritus. There is limited evidence of the efficacy of traditional Chinese therapies. We conducted a systematic review and meta-analysis to evaluate the efficacy of acupoint stimulation therapy in patients with uremic pruritus.

**Methods:**

A systematic search of seven databases (up to Sep 2022) was conducted for randomized controlled trials that evaluated the clinical efficacy of acupuncture, acupressure, auricular acupressure, acupoint injection, acupoint thermal therapy, acupoint sticking therapy, or transcutaneous electrical acupoint stimulation in the treatment of patients with uremic pruritus. Two reviewers selected eligible articles for inclusion in the meta-analysis and evaluated the risk of bias *via* Cochrane Collaboration. The results of pruritus assessments and uremic pruritus-related laboratory parameters were analyzed.

**Results:**

Forty trials published between 2002 and 2022, including a total of 2,735 participants, were identified for inclusion in the meta-analysis. The effective rates for acupuncture, auricular acupressure, and the combination of acupoint injection and acupoint massage were significantly greater in patients with uremic pruritus compared to the control group. The levels of serum BUN, PTH, and histamine levels were significantly lower vs. control group.

**Conclusions:**

Acupuncture, auricular acupressure, and the combination of acupoint injection and acupoint massage seem to be effective in improving uremic pruritus in patients with chronic kidney disease. However, further investigation of these potential treatments is now warranted in larger patient populations and over a longer time frame.

**Systematic review registration:**

https://www.crd.york.ac.uk/prospero/display_record.php?ID=CRD42022354585, identifier: PROSPERO CRD42022354585.

## Introduction

Uremic pruritus (UP) is a common cutaneous change that occurs in about 55% of patients with chronic kidney disease (CKD) who are undergoing dialysis (1). The manifestation of UP varies in severity, distribution, and duration, and UP can cause sleep disturbances, depressive symptoms, quality-of-life deterioration, and increased morbidity (2). Several pathophysiologic mechanisms and factors may be involved in UP, including inflammation, high serum calcium concentration, and histamine-dependent or non-histaminergic pruritogens, such as proteases, opioids, and substance P (3). Markers of dialysis inefficiency and mineral metabolism [e.g., high levels of phosphate, calcium, parathyroid hormone (PTH), and intact parathyroid hormone (iPTH)] may be associated with an increased risk of UP (4), for which treatments include topical, systemic, and immunomodulatory therapies (2); however, some UP patients remain refractory to traditional treatments (5).

Many complementary therapies have been evaluated in patients with UP, but with variable efficacy (5). Recently, traditional Chinese therapeutic methods, namely acupuncture, auricular acupressure (AA), acupoint massage (AM), acupoint injection (AI), and acupoint thermal therapy (ATT), demonstrated benefits in patients with UP. The benefit of acupuncture may be due to increased levels of anti-inflammatory cytokines, resulting from an altered type 1 to type 2 T-helper cell balance (6). Moreover, stimulation of Quchi (LI11) significantly reduced pruritus in patients with UP (7).

AI of autologous serum stimulates a non-specific immune response and decreases sensitivity to pruritus (8). Other medications, such as antihistamines, Gastrodin, Angelica, and Salvia, have also been administered as injections. For ATT, local acupoint infrared (AIR) radiation can improve nerve sensitivity and promote peripheral blood circulation, which may help patients with UP (9). Transdermal therapeutic administration of Chinese medicines at acupoints has also ameliorated pruritus (7).

Increased the number of the mast cells is one etiology for UP (7). Mast cells degranulation could release inflammatory markers such as histamine, tumor necrosis factors, and IL-6 (10). The activation of acupressure at LI11 can destroy mast cell which releasing inflammatory markers that might be the one etiology for UP and improve pruritus (7). Electroacupuncture (EA) at ST36 suppressing the activation of mast cells, leading to decrease of nerve growth factor (NGF) and tropomyosin receptor kinase A (TrkA) proteins (6). Acupuncture at ST36 can stimulate mast cell degranulation within the acupoint area and increase pain threshold (11). Interestingly, AA affects different parts of the body, by reconditioning the meridians, and is more easily accepted by patients (12). Transcutaneous electrical acupoint stimulation (TEAS) can also decrease the severity of UP (13), and the efficacy of TEAS is not statistically significantly different from that of acupressure (14). The combination of AI with acupressure (AI+A) is used to affect the endogenous opioid system, which might decrease anxiety and insomnia caused by pruritus (15).

To explore alternative treatments for UP, we performed a systematic review of several complementary therapies: acupuncture, AA, AM, AI, ATT, acupoint sticking therapy (AST), and TEAS.

## Methods

### Search strategy

We searched for articles published before 29 Sep 2022 from PubMed, Embase, CINAHL, Cochrane Library, China National Knowledge Infrastructure, Airiti Library, and Wanfang databases. The search string used was based on PubMed medical subject headings and Embase subject headings (Emtree): (chronic kidney disease OR kidney injury OR kidney failure OR chronic renal failure OR end-stage renal disease OR end stage renal disease OR dialysis OR hemodialysis OR peritoneal dialysis) OR (uremic OR uremia OR uremias) AND (pruritus OR itch OR xerosis OR skin problems OR skin disorders) AND (acupuncture OR acupressure OR Shiatsu OR Zhi Ya OR Chih Ya OR Shiatzu OR auricular acupuncture OR ear acupuncture OR auricular acupressure OR ear acupressure OR auricular therapy OR auriculotherapy OR auricular needle OR otopoint OR otoneedle OR auriculoacupuncture OR otopuncture OR acupressure point OR acupoints OR Tui Na).

We also searched for word combinations and free-text phrases containing the terms above and extended the search using the “related articles” function in PubMed. Further, all the retrieved abstracts, studies, and citations were reviewed. Finally, unpublished studies were obtained from the ClinicalTrials.gov registry (16). There were no language restrictions in the search. This systematic review and meta-analysis were registered online with PROSPERO, the international prospective register of systematic reviews of the National Institute for Health Research (ID: CRD42022354585) (17). The search protocol is attached in Supplementary material.

### Study selection

Randomized controlled trials (RCTs) were chosen to assess the efficacy of acupuncture, AA, AM, acupoint far infrared (AFIR), AI, AIR, AST, acupoint ion implantation, and transcutaneous electrical acupoint stimulation (TEAS) in the treatment of patients with UP. The inclusion criteria were: chronic kidney disease under dialysis; the presence of UP; administration of a specified treatment; and the availability of quantitative data for itch severity. If necessary, we contacted study authors for original or missing data. For studies with overlapping data, we selected those with the largest populations to exclude duplicate articles.

### Data extraction and quality assessment

Two reviewers independently screened the articles and extracted the following information: first author; publication date; participant characteristics; study design; inclusion, exclusion, and matching criteria; AA, AM, AFIR, AI, AIR, AST, or ATENS therapy; and quantitative data for itch severity. In line with the inclusion criteria, the two reviewers assessed the chosen articles for eligibility, and the reviewers' comments were recorded and compared. Any disagreements were submitted to, and reviewed by, a third investigator. Further, we performed a quality assessment of the studies using the “risk of bias” tool recommended by the Cochrane Collaboration (18). Several domains were evaluated, including method of allocation, blinding of participants and investigators, the integrity of outcome data, selective reporting, and other kinds of bias.

### Risk of bias assessment

#### Randomization bias

**Figure 1:**
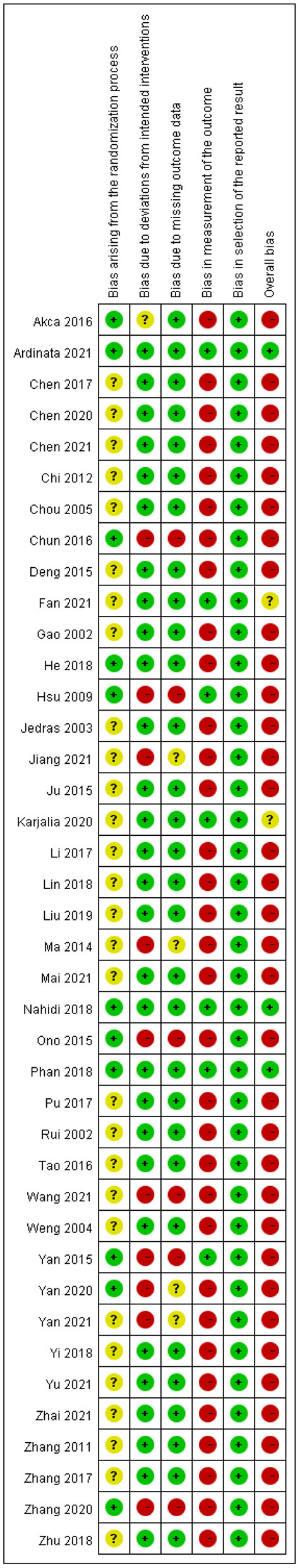
Flowchart showing the selection process for the studies included.

All 40 studies were RCTs: 10 studies provided no information about randomization; whereas 16 studies described the methods of randomization (nine studies used tables of random numbers, eight used random number generators, one used the drawing of lots, one used blocked randomization, and one study used chart numbers) (Figure 1).

#### Blinding

Seven studies were double-blind, and 33 studies were unblinded.

#### Incomplete outcome data

Eleven studies failed to use intention-to-treat analysis. The rate of loss to follow-up was low for most studies, although 10 studies had a dropout rate of more than 5%. Ten studies were also missing more than 5% of the outcome data and lacked evidence to support that data consistency was maintained despite the missing data. Six studies had other reasons for missing data, and 4 failed to report the reason for missing data.

### Data synthesis and analysis

We presented outcomes of the following tools for pruritus assessment to evaluate the efficacy of our chosen complementary therapies: visual analog scale (VAS) and numeric rating scale (NRS; 0–10 points for each scale); 5-dimensional itch scale (5DIS: 5–25 points, and comprising dimensions of degree, duration, direction, disability, and distribution of itching, with 1–5 points for each dimension); the modified Duo's pruritus score (mDuo; 0–40 points); four-item itch questionnaire [FIIQ; evaluations of pruritus localization (1–3 points), severity (1–5 points), frequency (1–5 points), and sleep disturbances due to itching (0–6 points); total 3–19 points]; Pauli-Magnus scale (PMS; 3–45 points); and Dirk R Kuypers score (DRKS; 27 points); Traditional Chinese medicine symptom scale, UP questionnaire, 12-Item Pruritus Severity Scale (12-PSS; 3–22) (19) and Serjip score (Assessment of severity and burden of pruritus) (20).

The statistical package Review Manager (version 5; Cochrane Collaboration, Oxford, England) was used for data analysis. A meta-analysis was conducted based on recommendations outlined in the Preferred Reporting Items for Systematic Reviews and Meta-Analyses (PRISMA) guidelines. Standard deviations (SDs) were calculated from the provided confidence interval (CI) limits, standard errors, or ranges when necessary. We obtained the mean and SD from studies using the mean difference (MD) or standardized mean difference (SMD) with 95% CIs for continuous outcomes. The random-effects model was applied to pool estimates of SDs and SMDs, considering the diversity of pruritus assessment tools and possible heterogeneity across the trials. We considered heterogeneity in the studies by performing the *I*^2^ test and a null hypothesis test, in which p<0.05 confirmed significant heterogeneity among the outcomes. The Guideline Development Tool developed by the Grading of Recommendations Assessment, Development and Evaluation Working Group was applied to assign the quality of the evidence (21). All data generated or analyzed during this study are included in this published article (and its Supplementary Information files).

## Results

### Literature search

**Figure 2:**
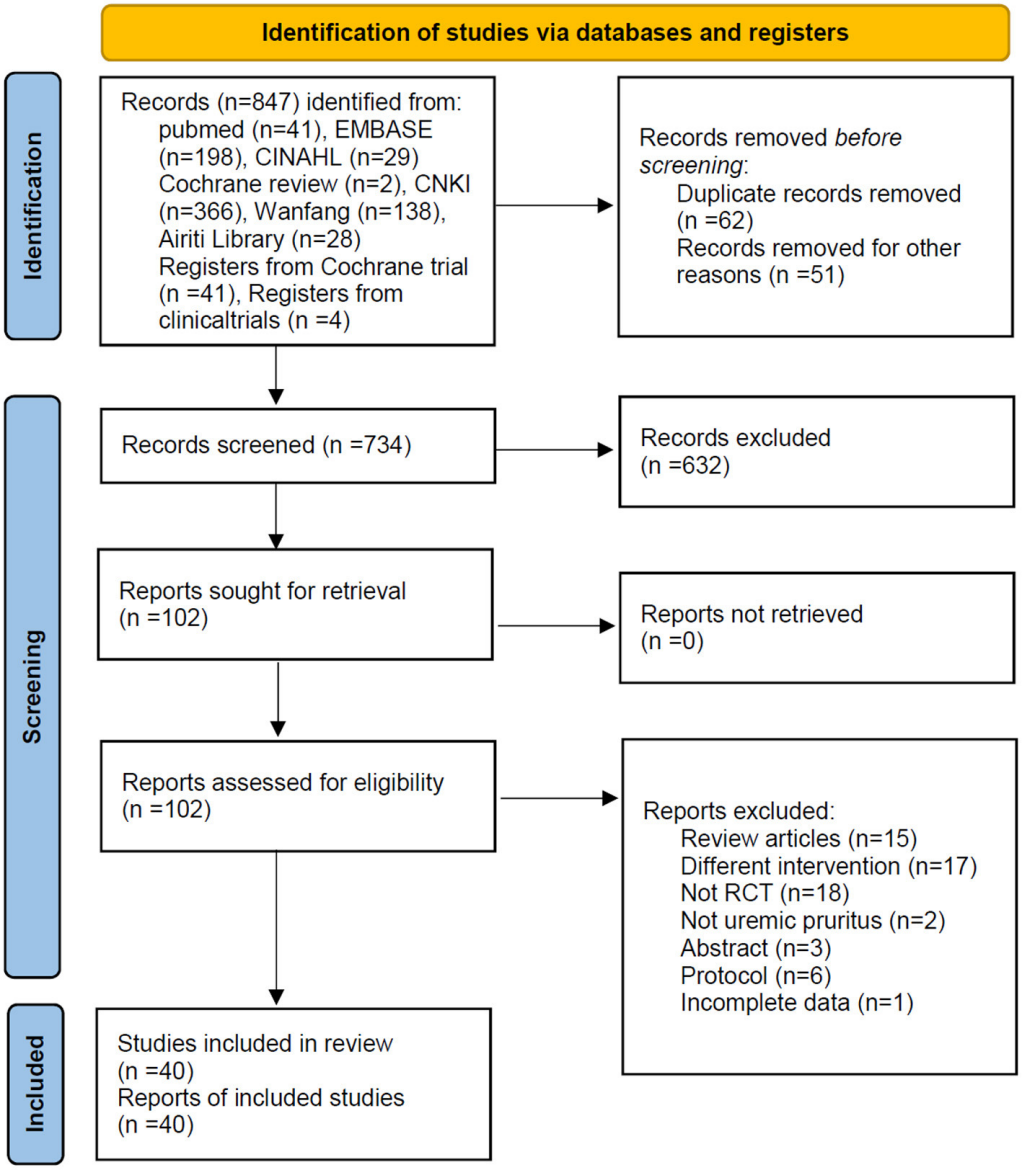
Risk of bias in the selected studies.

The selected studies were all RCTs from 2002 to 2022. A total of 2,735 patients were enrolled in the 36 studies (Figure 2). In total, we filtered out 847 citations: 62 duplicate articles, and 51 articles due to other reasons, were removed by EndNote and the reviewers; further, 734 articles were excluded following the screening criteria for titles and abstracts. We retrieved the full texts of the remaining 102 articles. After assessing for eligibility, we excluded 62 papers because 15 were reviews, 17 papers were studies conducted in patients undergoing treatments other than our chosen interventions, 18 papers were non-randomized studies, 2 papers did not focus on UP, 3 papers were conference abstracts, 6 papers were presented in the protocol, and 1 paper had incomplete data. After screening, 40 articles fulfilled the selection criteria and were included in the meta-analysis.

### Study characteristics

Data for treatment frequency, duration, acupoint, the pruritus severity assessment tool, corresponding results, and follow-up duration are shown in Table 1. There were 16 studies in the acupuncture group, 13 studies in the AA group, 4 studies in the AM group, 1 study in the AI group, 1 study in the AST group, 1 study in the AFIR group, and 1 study in the ATENS group. There were 3 studies of combination therapies: two of AI+acupuncture; and one of AI+AM. Thirteen studies reported effective rates. Twenty studies reported pruritic scores including data on the VAS, mDuo, 5DIS, NRS, PMS, traditional Chinese medicine (TCM) symptom score, FIIQ, Serjip score, UP questionnaire, and DRKS.

**Table 1:** Characteristics of included studies.

**Study (year)**	**Study design**	**Inclusion criteria**	**No. of patients**	**Age (year-old)**	**Treatment (route, dosage, and frequency)**	**Control (route, dosage, and frequency)**	**Concomitant** ** treatment**	**Duration**	**Inspection data**	**Pruritus severity assessment tool**	**PS (before → after), experimental control**	**Effective rate**	**Follow up duration**	**Acupoint**
**Acupuncture**														
Ardinata et al. (22)	RCT	HD	T: 30 C:30	T: – C: 25~77	A (3 times/W) HD	Placebo + HD	Conventional Tx	12 W	IL-31	5DIS	T: 1 (1–5) → 1 (1–5) 4 (2–5) → 2 (1–4) 4 (2–5) → 3 (2–4) 3 (1–5) → 2 (1–4) C: 2 (1–5) → 1.5 (1–5) 4 (2–5) → 3 (1–5)	NA	NA	LI11
Fan et al. (23)	RCT	HD	T: 22 C:20	T: 62.4 (4.10) C: 63.2 (4.50)	A (3 times/W) HD	Conventional western medicine	Conventional Tx	12 W	Ca, P, BUN, Cr	Grade, distribution, sleep	T: 38.20 (4.80) → 17.30 (5.50) C: 38.30 (4.30) → 37.50 (3.20)	NA	NA	LI11
Jiang et al. (24)^†^	RCT	HD	T1 (hemoperfusion): 14 T2 (hemoperfusion + A): 14 C: 14	T1: 72.43 (7.56) T2: 73.50 (7.77) C: 72.93 (9.09)	Hemoperfusion A (3 times/W) HD	Placebo + HD	Conventional Tx	NA	Ca, P, iPTH, hs-CRP, IL-6	VAS 5DIS	T1: 2 (3.70) → 1 (3.29) T2: 1.5 (3.08) → 0 (0.61) → 0 (0.61) C: 2.00 (3.70) T1: 13.5 (8.64) → 8.5 (5.76) T2: 13.5 (7.00) → 6.5 (4.11) C: 9.5 (8.44) → 10 (8.23)	NA	NA	LI4 LI11 SP6 SP10 ST36
Zhang et al. (25)	RCT	HD	T: 30 C: 33	T: 53.63 (9.40) C: 52.23 (7.95)	A (3 times/W) + HD	HD	Conventional Tx	4 W	CRP, Hb, albumin, BUN, Cr, Ca, P, PTH, Eosinophil, IgE	VAS	^Δ^T: 5 (3–8) ^Δ^C: 2 (−1–4)	T: 13/35 C: 2/35	NA	LI11 SP10 SP6
										mDUO	^Δ^T: 6.23 (4.83) ^Δ^C: 1.73 (2.82)			
										TCM syndrome score	^Δ^T: 9 (7–11) ^Δ^C: 0 (−0.5–2)			
Liu et al. (26)	RCT	HD	T: 40 C: 40	T: 54.80 (3.20) C: 53.50 (4.60)	A (3 times/W) + HD	HD	Conventional Tx	4 W	Ca, P, PTH, histamine	Distribution, frequency, severity, sleep	Distribution T: 2.45 (0.87) → 1.41 (0.52) C: 2.49 (0.83) → 2.34 (0.78) Frequency T: 3.69 (1.21) → 1.52 (0.52) C: 3.72 (1.32) → 3.63 (1.53) Severity T: 3.59 (1.12) → 1.32 (0.45) C: 3.61 (1.14) → 3.21 (1.43) Sleep T: 7.33 (2.14) → 2.14 (0.89) C: 7.45 (2.18) → 7.23 (2.09)	NA	NA	LI11 SP6
Nahidi et al. (27)	RCT	HD	T: 15 C: 11	T: 54.67 (11.40) C: 41.36 (16.21	A (3 times/W) + HD	Placebo + HD	Conventional Tx	6 W	NA	VAS	T: 9.87 (0.35) → 3.93 (2.85) C: 9.45 (0.93) → 8.18 (1.40)	NA	NA	SP6 SP10 LIV3 LI4
Phan et al. (28)	RCT	HD	T: 18 C: 19	T: 45.72 (10.08) C: 48.37 (10.81)	A (2 times/W) + HD	Placebo + HD	Conventional Tx	6 W	NA	5DIS	T: 12.00 (3.27) → 7.89 (0.83) C: 12.74 (3.07) → 10.63 (3.17)	NA	8 W	LI11 Quchi
Chu et al. (29)	RCT	HD	T: 20 C: 20	T: 44.80 (11.50) C: 45.50 (12.00)	A (2 times/W) + HD	Loratidine (10 mg QD) +HD	Conventional Tx	12 W	BUN, Cr, P, PTH, β2-MG	VAS	T: 8.65 (1.24) → 1.45 (0.58) C: 8.60 (1.18) → 5.24 (1.28)	T: 19/20 C: 13/20	NA	SP10 ST36 SP6 LI4 LI11 DU20
										VAG	T: 7.52 (0.64) → 2.80 (0.65) C: 7.38 (0.45) → 5.75 (1.85)			
Pu et al. (30)	RCT	HD	T: 27 C: 27	T: 63.52 (5.18) C: 63.42 (5.32)	A (2 times/W) + HD	HD	Conventional Tx	10 D	NA	Dirk. R. Kuypers scale (DRKS)	T: 25.5 (5.3) → 10.4 (3.4) C: 25.2 (5.1) → 18.9 (4.1)	T: 24/27 C: 20/27	NA	LI4 LI11 ST36 LU5
Chang et al. (31)	RCT	HD	T1 (A): 16 T2 (citrate): 17 C: 17	Total: 45.30 (18.90)	T1: A + HD T2: citrate dialysate	HD	Conventional Tx	Not mentioned	CRP, WBC	Guidelines for clinical research of Traditional Chinese Drug Research	T1: 12.12 (1.78) → 9.16 (1.67) T2: 12.17 (1.75) → 4.08 (1.84) C: 12.08 (1.82) → 10.80 (1.96)	T1: 9/16 T2: 15/17 C: 2/15	NA	LI11 GB31 ST36 SP10 BI17
Ono et al. (32)	P-RCT	HD	T: 24 C: 23	T: 70.00 (9.60) C: 67.30 (13.00)	A (1 time/W) + HD + M-test	HD	T: 0.66 (0.15) → 0.76 (0.17) C: 0.64 (0.18) → 0.64 (0.18)	8 W	Ca, P, Hb, Cr, BUN, albumin, leukocyte	NRS	60 → 18	NA	12 W	LU5 LU9 LI2 LI11 HT7 HT9 SI3 SI8 PC7 PC9 TE3 TE10 SP2 SP5 ST41 ST45 KI1 KI7 BL65 BL67 LR2 LR8 GB38 GB43
										Utility				
Ma et al. (33)	RCT	HD	T: 23 C: 23	T: 64.33 (13.77) C: 60.74 (16.36)	A (3 times/W) + HD	HD	Conventional Tx	16 W	NA	Clinical effect	NA	T: 18/23 C: 1/19	NA	SP10 LI4
										mDuo	T: 14.96 (5.89) → 4.35 (4.74) C: 19.45 (8.71) → 20.21 (8.48)			
Chang et al. (34)	RCT	HD	A+HDF: 15 HDF: 15 C (HD): 16	A+HDF: 52.30 (12.60) HDF: 49.60 (13.40) C: 53.20 (15.90)	A (2 times/W) + HD + HDF	HDF and HD	Conventional Tx	12 W	P, PTH	NA	NA	A+HDF: 13/15 HDF: 10/15 C: 3/16	NA	LI4 LI11 ST36 SP10 LU5 BI17
										FIIQ	T: 13.03 (1.96) → 8.56 (1.76) C: 12.22 (2.10) → 10.08 (1.84)			
Yu et al. (35)	RCT	HD	T: 50 C: 50	T: 55.60 (1.90) C: 53.60 (2.50)	Auricular scraping (1 time/W)	Loratidine 10 mg/d	Conventional Tx	4 W	NA	VAS FIIQ	NA T: 8.42 (3.30) → 5.41 (2.25) C: 8.48 (4.21) → 6.79 (3.02)	T: 43/50 C: 38/50	8 W	Lung Large intestine Shenmen (TF4) Endocrine (CO18) Adrenal gland Occiput
Zhai et al. (36)	RCT	HD	T: 50 C: 50	T: 62.98 (3.65) C: 63.23 (3.92)	AA (4 times/d) + HD	Loratadine (10 mg QD) + HD	Conventional Tx	12 W	NA	PSQI	T: 20.21 (2.15) → 13.67 (2.97) C: 20.34 (2.72) → 17.13 (2.12)	Itching: T: 46/50 C: 38/50	NA	Kidney Spleen Stomach Sympathy Subcortical TF4
Che et al. (5)	RCT	HD	T: 20 C: 20	T: 62.40 (9.10) C: 63.20 (7.50)	A (2 times/W) + HD	Placebo needle + HD	Conventional Tx	4 W	NA	severity, distribution, sleep disturbance questionnaire	T: 38.20 (4.80) → 17.30 (5.50) C: 38.50 (3.20) → 37.50 (3.20)	NA	12 W	SP6 SP10 ST36 LI11
Ruei et al. (37)	RCT	HD	T: 80 C: 70	T: 21–73 C: 24–69	A (2 times/W or 3 times/2W) + HD	Calcitrol (2 ug two times/W or 3 times/2W) + HD	Conventional Tx	16 W	NA	NA	NA	T: 71/80 C: 62/80	12 M	LI11 ST36 SP6 SP10
Kao et al. (38)	RCT	HD	T: 34 C: 34	Total 22~72 (mean 43.6)	A (2 times/W) + HD	Chlorpheniramine + HD	Conventional Tx	4 W	NA	NA	NA	T: 33/34 C: 24/34	NA	LI11 ST36
**Auricular acupressure (AA)**														
Mai et al. (39)	RCT	HD	T: 40 C: 40	T: 45.70 (15.20) C: 44.97 (17.30)	AA (3 times/d) + HD	HD	Conventional Tx	4 W	QLQ-C30 (life quality)	Pauli-Magnus scale	T: 25.23 (16.42) → 3.75 (1.42) C: 24.85 (17.67) → 5.65 (1.67)	NA	NA	Lung Heart Endocrine Lung Heart Endocrine SF1.2i
Yan et al. (40)	RCT	HD	T: 32 C: 36	T: 57.72 (12.16) C: 61.06 (11.77)	AA (9 times/2ds) + HD	HD	Conventional Tx	12 W	Ca, P, PTH, IL-2, IL-6, IL-8, IL-10	VAS	T: 5.69 (0.97) → 2.66 (0.87) C: 5.75 (0.94) → 4.19 (0.92)	NA	NA	Kidney Lung Heart Endocrine Subcortical TF4
Chen et al. (41)	RCT	HD	AA+ND: 30 ND: 30 C: 30	AA+ND: 44.10 (1.60) ND: 43.90 (1.40) C: 44.20 (1.50)	ND (3 times/W) AA+ND: AA (3~5 imes/d) + ND	HD	Conventional Tx	48 W	Ca, P, PTH, ALP, vitamin D	NA	NA	Itching: AA+ND: 20/30 ND: 8/30 C: 5/30	NA	TF4 Kidney Spleen Stomach Subcortical Sympathy
Yan et al. (42)	RCT	HD	T: 39 C: 39	T: 57.16 (17.01) C: 54.40 (8.37)	AA (9 times/2ds) + HD	HD	Conventional Tx	12W	Ca, P, PTH, amylase, histamine, IL-2	VAS	T: 5.84 (1.82) → 3.88 (1.50) C: 5.84 (2.06) → 5.37 (1.90)	NA	NA	Kidney Lung Heart Endocrine Subcortical TF4
Ding et al. (43)	RCT	PD	Total: 65 T: – C: –	T: 60.76 (12.33) C: 64.42 (10.47)	AA (8 times/2ds) + HD	HD	Conventional Tx	12 W	Ca, P, PTH, Hb, CRP, histamine, IL-2, IL-6, IL-8, IL-10	VAS	T: 5.29 (0.97) → 2.24 (0.78) C: 5.42 (1.12) → 4.10 (1.01)	NA	NA	Kidney Lung Heart Endocrine Subcortical TF4
										FIIQ	T: 11.71 (2.34) → 8.18 (1.70) C: 12.81 (2.27) → 10.29 (1.90)			
										TCM symptom score	T: 34.12 (5.89) → 25.94 (2.93) C: 36.13 (5.32) → 30.32 (4.82)			
He et al. (44)	RCT	HD	T: 34 C: 35	T: 54.18 (10.76) C: 50.23 (12.69)	AA (4~5 times/2ds) + HD	HD	Conventional Tx	4 W	Ca, P, PTH, Cr, BUN, albumin, Hb	Pauli-Magnus scale	Scratching activity T: 2.74 (1.33) → 1.18 (0.39) C: 2.54 (0.85) → 2.14 (0.69) Distribution of pruritus T: 1.94 (0.78) → 1.06 (0.24) C: 1.63 (0.73) → 1.60 (0.60) Awaking from itching T: 1.53 (2.87) → 0.12 (0.48) C: 1.60 (1.80) → 0.86 (1.00) Number of nighttime scratching T: 2.24 (1.52) → 0.65 (0.88) C: 1.83 (1.07) → 1.80 (1.08)	NA	NA	Lung Endocrine Adrenal
										VAS	T: 4.59 (2.03) → 1.79 (1.10) C: 3.80 (1.39) → 3.29 (1.13)			
Lin et al. (45)	RCT	CKD	Fumigation: 30 AA: 30 Fumigation + AA: 30 C: 30	NA	AA (3~5 times/2ds) (Fumigation ingredient: Tufulin, Huangba, Kushen, Machixian, Licorice, Senecio)	Conventional Tx	Conventional Tx	8 W	P, PTH	Serjip score	Fumigation: 326 → 116 AA: 325 → 126 Fumigation+AA: 336 → 136 C: 316 → 207	NA	NA	Lung Spleen Stomach Adrenal supracortical Sympathy Endocrine Wheel area
Li et al. (46)	RCT	HD	T: 40 C: 40	T: 53.80 (3.60) C: 55.10 (1.0.80)	AA (4 times/d) + HD	Acrivastine (8 mg 3 times/d) + HD	Conventional Tx	4 W	NA	NA	NA	T: 38/40 C: 28/40	NA	A: Kidney Heart Lung Liver spleen Sanjio B: Bladder SHENMEN HX1 HX6.7i SF1.2i AT2.3.4i
Tao et al. (47)	RCT	HD	T: 40 C: 40	T: 55.90 (3.10) C: 56.20 (3.50)	AA (5~8 times/d) + HD	HD	Conventional Tx	8~12 W	NA	itching severity, frequency, sleep	T: 19.36 (2.37) → 7.36 (2.18) C: 19.71 (2.33) → 13.27 (2.23)	NA	NA	Kidney Spleen Stomach Sympathy Subcortical Shenmen (TF4)
Yan et al. (12)	RCT	HD	T: 32 C: 30	Total 20~65	AA (5~8 times/d) + HD	Placebo + HD	Conventional Tx	6 W	Ca, P, PTH, histamine, substance P, PAR-2, tryptase	VAS	T: 5.75 (2.03) → 3.84 (1.69) C: 5.60 (2.13) → 5.57 (2.29)	NA	NA	Shenmen (TF4), kidney (CO10), lung (CO14), endocrine (CO18), subcortical (AT4)
Shr et al. (48)	RCT	HD	T: 30 C1 (HD+HP): 30 C2 (HD): 30	Total 58.00 (17.00)	AA (>4 times/d) + HD	HD+HP or HD	Conventional Tx	4 W	Ca, P, PTH, BUN, β2-MG, Cr	NA	NA	T: 26/29 C1: 23/27 C2: 10/28	NA	A: Heart Lung Liver Spleen Sanjiao B: Bladder Shenmen (TF4)
**Acupoint far infrared (AFIR)**														HX1 HX6.7i SF1.2i AT2.3.4i
Hsu et al. (49)	RCT	HD	T: 21 C: 20	T: 57.14 (2.74) C: 66.90 (3.06)	AFIR (1 time/d, 2 ds/W)	HD	Conventional Tx	18 W	Ca, P, albumin, Urea, ALK-P, Hb, PTH	Uremic pruritus questionnaire	T: 17.85 (2.33) → 6.43 (0.91) C: 17.55 (2.00) → 9.05 (1.59)	NA	NA	SP6
										VAS	T: 18.57 (1.41) → 10.71 (1.17) C: 16.50 (1.35) → 10.70 (1.03)			
**Acupoint injection (AI)**														
Wang et al. (50)	RCT	HD	T: 55 C: 54	T: 56.40 (8.60) C: 56.40 (8.80)	AI (2 times/W) + Neurotin (0.1 g 2 times/d) + HD	Neurotin + HD	Conventional Tx	4 W	Ca, P, PTH	VAS	T: 6.43 (1.24) → 3.35 (1.52) C: 6.25 (1.22) → 4.38 (1.19)	NA	NA	LI11 ST36
										DRKS	T: 10.96 (3.40) → 5.46 (2.17) C: 12.16 (9.55) → 7.60 (2.63)			
**Acupoint injection and acupuncture (AI** + **acupuncture)**														
Deng et al. (51)	RCT	HD	T: 23 C: 23	Total 42.73 (3.10)	AI (2 times/W) + acupuncture + cetirizine (10 mg QHS)	HD	Conventional Tx	12W	NA	NA	NA	T: 22/23 C: 19/23	NA	AI + A: LI11 ST36 SP10
Wang et al. (52)	RCT	HD	T: 56 C: 54	T: 29~78 C: 25~69	AI + acupuncture (2 times/W or 3 times/2 Ws) + HD	Calcitrol (0.25 ug 1 time/d or 1 time/2ds) + HD	Conventional Tx	12 W	NA	NA	NA	T: 51/56 C: 48/54	NA	A: EM40 GB31 LI4 LI11 ST36 SP6 SP9 SP10 AI: BI17 BI23
**Acupoint injection** + **acupoint massage (AI** + **AM)**														
Chen et al. (53)	RCT	HD	T: 30 C: 30	T: 49.17 (12.20) C: 49.77 (1.24)	AI (2~3 times/W) + AM (2~3 times/W) + HD	HD	Conventional Tx	8 W	NA	VAS	T: 7.50 (1.11) → 5.23 (1.36) C: 7.33 (1.32) → 7.27 (1.36)	T: 27/30 C: 20/30	NA	AI: LI11 ST36 AM: DU14 DU23 EM1 EM2 HT7 GB20 LI4 SP6 SP10 PC4
**Acupoint infrared (AIF)**														
Yi et al. (9)	RCT	HD	T: 20 C: 20	T: 56.37 (4.22) C: 56.89 (4.19)	AIR (2 times/W) + HD	HD	Conventional Tx	5 W	Ca, P, PTH, albumin, BUN, Cr, Hb, WBC, Plt,	VAS	T: 7.82 (1.69) → 4.17 (2.86) C: 7.49 (1.30) → 6.02 (2.15)	NA	NA	LI11 SP6 SP10
										Sleep quality score	T: 1.92 (0.78) → 0.72 (0.45) C: 1.88 (0.85) → 1.68 (0.91)			
**Acupoint massage (AM)**														
Chen et al. (54)	RCT	High-flux HD	T: 31 C: 31	T: 47.90 (13.40) C: 48.30 (11.20)	AM (3 times/W) High-flux HD (3 times/W)	High-flux HD	Conventional Tx	8 W	P, iPTH, hs-CRP	12-PSS	T: 18.40 (4.20) → 11.60 (3.20) C: 19.10 (6.30) → 13.80 (3.40)	NA	NA	LI11
Karjalian et al. (7)	RCT	HD	T: 30 C1 (Placebo massage): 30 C2 (no intervention: 30	T: 55.31 (8.88) C1: 52.67 (10.89) C1: 55.00 (11.14)	AM (3 times/W)	C1 (Placebo massage, 3 times/W) + HD C2 (HD)	Conventional Tx	4 W	Ca, P, Na, K, PTH, BUN, Cr, Hb	NRS	T: 8.37 (1.22) → 2.87 (0.90) C1: 7.67 → 6.37 C2: 7.73 → 7.33	NA	5 W	SP6, SP10 ST36 LI11
Akca et al. (14)	RCT	HD	AM: 25 ATENS: 24 C: 25	AM: 55.24 (10.13) TEAS: 48.08 (9.05) C: 45.84 (10.40)	AM (3 times/W) ATENS (3 times/W)	HD	Conventional Tx	4 W	NA	VAS	AM: 6.84 (1.70) → 3.36 (2.37) ATENS: 7.37 (1.31) → 3.12 (2.15) C: 6.92 (1.41) → 5.08 (1.55)	NA	NA	ATENS: LI-11
Jedras et al. (55)	RCT	HD	T: 30 C: 30	T: 46.63 (12.41) C: 44.57 (10.71)	AM (3 times/W)	HD	Conventional Tx	5 W	NA	frequency, intensity localization, influence on wellbeing questionnaire	T: 8.53 (2.31) → 1.07 (1.91) C: 7.70 (2.30) → 7.57 (2.03)	NA	18 W	140 pressure points (20 each on the head, hands, trunk, legs)
**Acupoint sticking therapy (AST)**														
Jiu et al. (56)	RCT	HD	T1 (HD): 20 T2 (HD+HPF): 20 T3 (HD+HF): 20 C1 (HD): 20 C2 (HD+HPF): 20 C3 (HD+HF): 20	T1: 45.20 (1.90) T2: 44.60 (2.00) T3: 45.30 (1.80) C1: 45.30 (2.10) C2: 44.70 (2.70) C3: 45.1 (2.20)	AST (Difuzi, Tusizi, Mudanpi, and Taoren/2ds)	HD, HPF, HF	Conventional Tx	12 W	Ca, P, PTH, Hb, BUN, Cr	VAS	T1 vs C1 T1: 5.95 (2.26) → 5.20 (2.49) C1: 6.65 (1.57) → 5.05 (1.64) T2 vs C2 T2: 6.85 (1.56) → 3.20 (1.08) C2: 6.40 (1.78) → 3.75 (1.12) T3 vs C3 T3: 6.90 (1.80) → 1.80 (2.03) C3: 5.85 (1.89) → 2.30 (2.03)	NA	NA	Umbilicus
**Transcutaneous electrical acupoint stimulation (TEAS)**														
Akca et al. (14)	RCT	HD	AM: 25 TEAS: 24 C: 25	AM: 55.24 (10.13) ATENS: 48.08 (9.05) C: 45.84 (10.40)	AM (3 times/W) ATENS (3 times/W)	HD	Conventional Tx	4 W	NA	VAS	AM: 6.84 (1.70) → 3.36 (2.37) ATENS: 7.37 (1.31) → 3.12 (2.15) C: 6.92 (1.41) → 5.08 (1.55)	NA	NA	ATENS: LI-11

RCT, randomized controlled trial; T, treatment group; C, control group; Tx, treatment; NA, not applicable; HD, hemodialysis; HDF, hemodiafiltration; HF, hemofiltration; HP, hemoperfusion; ND, nocturnal dialysis; AA, auricular acupressure; ATENS, Acupoint transcutaneous electrical nerve stimulation; W, week; d, day; 5DIS, 5-D itch scale; VAS, visual analog scale; DRKS, Dirk R. Kuypers scale; TCM syndrome score, traditional Chinese medicine syndrome score; PSQI, Pittsburgh Sleep Quality Index; 12-PSS, 12-Item Pruritus Severity Scale; PS, performance status.

Δ Difference between two numbers.

† Median (interquartile range).

All studies included patients with UP and CKD: in 38 studies, patients received regular hemodialysis, while in the Ding et al. and Chen et al. studies, patients underwent peritoneal dialysis and nocturnal dialysis, respectively (41, 43). There were 16 studies on acupuncture. The treatment frequency was about two to three times per week. The most common acupoint choices were Xuehai (SP10, 9 studies), LI11 (7 studies), Sanyinjiao (SP6), and Zusanli (ST36, each of these acupoints used in 7 studies). Chang et al. added citrate dialysate as UP therapy and the result revealed improved pruritus (31).

There were 13 studies of AA, and the treatment frequency was about three to eight times per day. The meridian in these studies included lung, kidney, heart, stomach, endocrine, subcortical, supracortical, adrenal, and Shenmen (TF4). Chen et al. also compared nocturnal dialysis with hemodialysis (41), and Lin et al. compared the efficacy of AA, fumigation, and the combination of AA+fumigation therapy (45), and Yu et al. used copper scraping therapy at auricular acupoint (35).

There was one study of AFIR, and the treatment frequency was once per day, on 2 days each week. The acupoint used for AFIR was SP6. There was one study of AI, for which the treatment frequency was twice per week. Wang et al. used gabapentin as a control arm, and the acupoints used were LI11 and ST36 (50). There was one study of AIR, in which the treatment frequency was twice per week, and in which the acupoints used were LI11, SP6, and SP10. There were four studies of AM, with a treatment frequency of three times per week, and with acupoints of SP6, SP10, ST36, and LI11. Chen et al. compared high-flux HD with AM+high flux HD in 12-PSS score (54). There was one study of AST, in which the treatment frequency was once every 2 days: that is, Jiu et al. used Di Fu Zi, Tu Si Zi, Mud An Pi, and Tao Ren as TCMs in sticking therapy applied at the umbilicus (56).

Four studies evaluated complementary-therapy combinations: AI+A (*n* = 2), AI+AM (*n* = 1), and AM+TEAS (*n* = 1). Deng et al. assessed AI+A with cetirizine and treated patients twice each week; the acupoints used were LI11, ST36, and SP10 (51). Wang et al. assessed AI+AM twice each week, or three times every 2 weeks; the acupoints used were Jianneiling (EM40), Fengshi (GB31), Hegu (LI4), LI11, ST36, SP6, Yinlingquan (SP9), and SP10 for AM, and Geshu (BI17) and Shenshu (BI23) for AI (52). Chen et al. evaluated AI+AM two to three times per week to treat UP; the acupoints used for AI were LI11 and ST36, and those used for AM were Dazhu (DU14), Shangxing (DU23), Sishencong (EM1), Yintang (EM2), Shenmen (HT7), Fengchi (GB20), LI4, SP6, SP10, and Ximen (PC4) (53).

### Pruritus assessments

#### Visual analog scale (VAS) score

**Figure 3:**
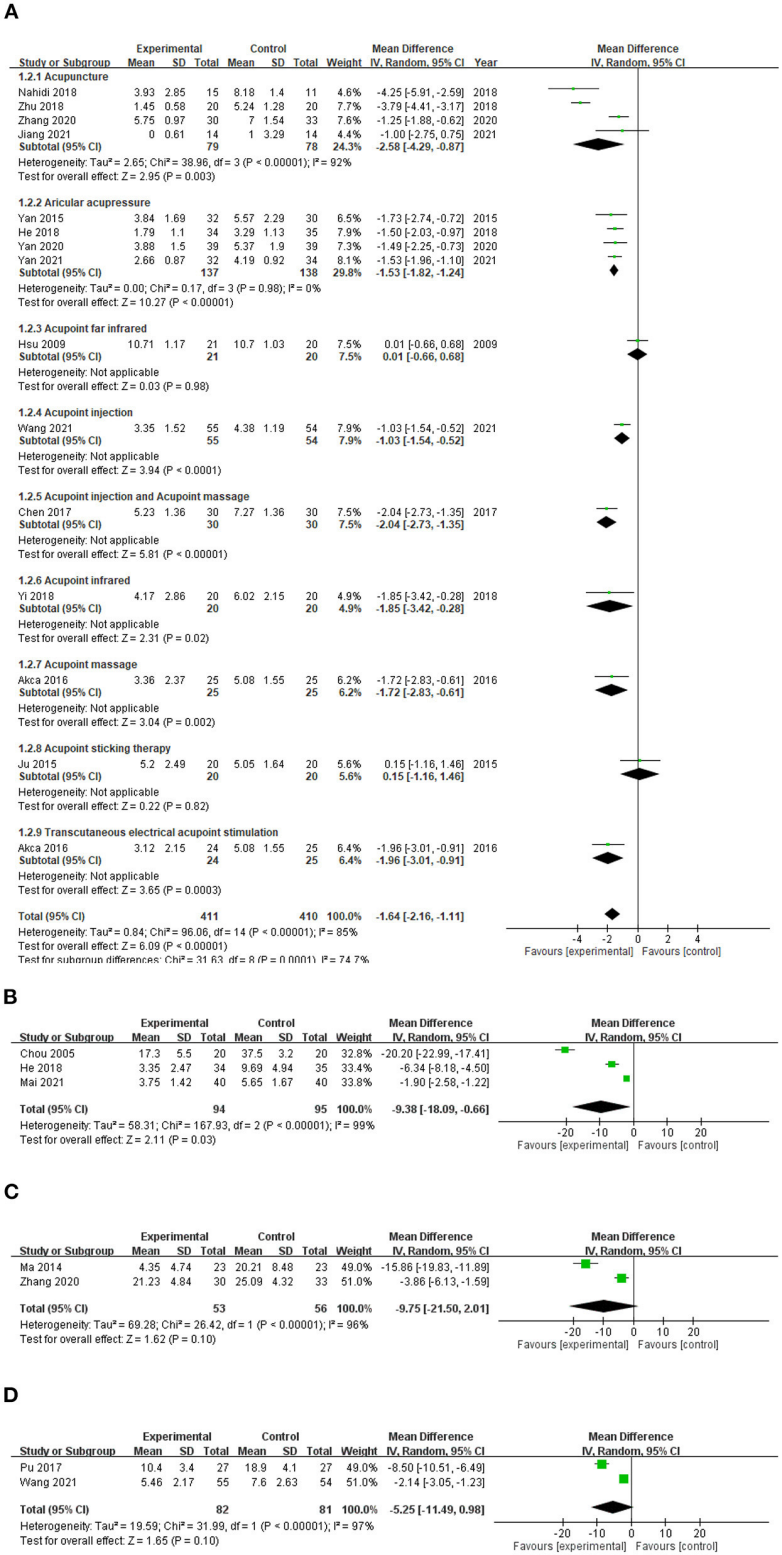
Forest plot comparison of pruritus scores on **(A)** visual analog scale, **(B)** Pauli-Magnus scale, **(C)** modified Duo's pruritus score, and **(D)** Dirk R. Kuypers scale in patients with uremic pruritus treated with acupuncture. CI, confidence interval; df, degrees of freedom; IV, independent variable; SD, standard deviation.

Meta-analysis of the 15 studies with VAS score assessments (Figure 3A) showed statistically significant experimental–control group improvements in VAS score for acupuncture (MD −2.58; 95% CI: 4.29, −0.87; *p* = 0.003), AA (MD −1.53; 95% CI: −1.82, −1.24; *p* < 0.00001), AI (MD −1.03; 95% CI: −1.54, −0.52; *p* < 0.0001), AI+AM (MD −2.04; 95% CI: −2.73, −1.35; *p* < 0.00001), AIR (MD −1.85; 95% CI: −3.42, −0.28; *p* = 0.02), AM (MD −1.72; 95% CI: −2.83, −0.61; *p* = 0.002), and TEAS (MD −1.96; 95% CI: −3.01, −0.91; *p* = 0.0003). No statistically significant improvement was evident for AFIR (MD 0.01, 95% CI: −0.66, 0.68; *p* = 0.98) or AST (MD −0.42; 95% CI: −0.96, 0.13; *p* = 0.13). Nonetheless, for all 15 studies combined, a significant decline in VAS score was evident in the experimental vs. control group (MD −1.64; 95% CI: −2.16, −1.11; *p* < 0.00001), with significant heterogeneity among the studies (*I*^2^ 86%; *p* < 0.00001).

#### Pauli-Magnus scale (PMS) score

Significant experimental–control group decreases in PMS scores (Figure 3B) were also noticed in three studies that included a total of 189 patients with UP (overall MD −9.38; 95% CI: −18.09, −0.66; *p* = 0.03), with significant heterogeneity among the studies (*I*^2^ 99%; *p* < 0.00001).

#### Modified Duo's (MDuo) pruritus score

Two studies in a total of 109 patients with UP showed no statistically significant overall improvement in mDuo score (Figure 3C) in the experimental vs. control group (MD −9.75; 95% CI: −21.50, +2.01; *I*^2^ 96%; *p* = 0.10).

#### Dirk R. Kuypers scale (DRKS) score

Two studies in a total of 163 patients with UP showed no statistically significant overall improvement in DRKS score (Figure 3D) in the experimental vs. control group (MD −5.25; 95% CI: −11.49, +0.98; *I*^2^ 97%; *p* = 0.10).

#### Effective rate

**Figure 4:**
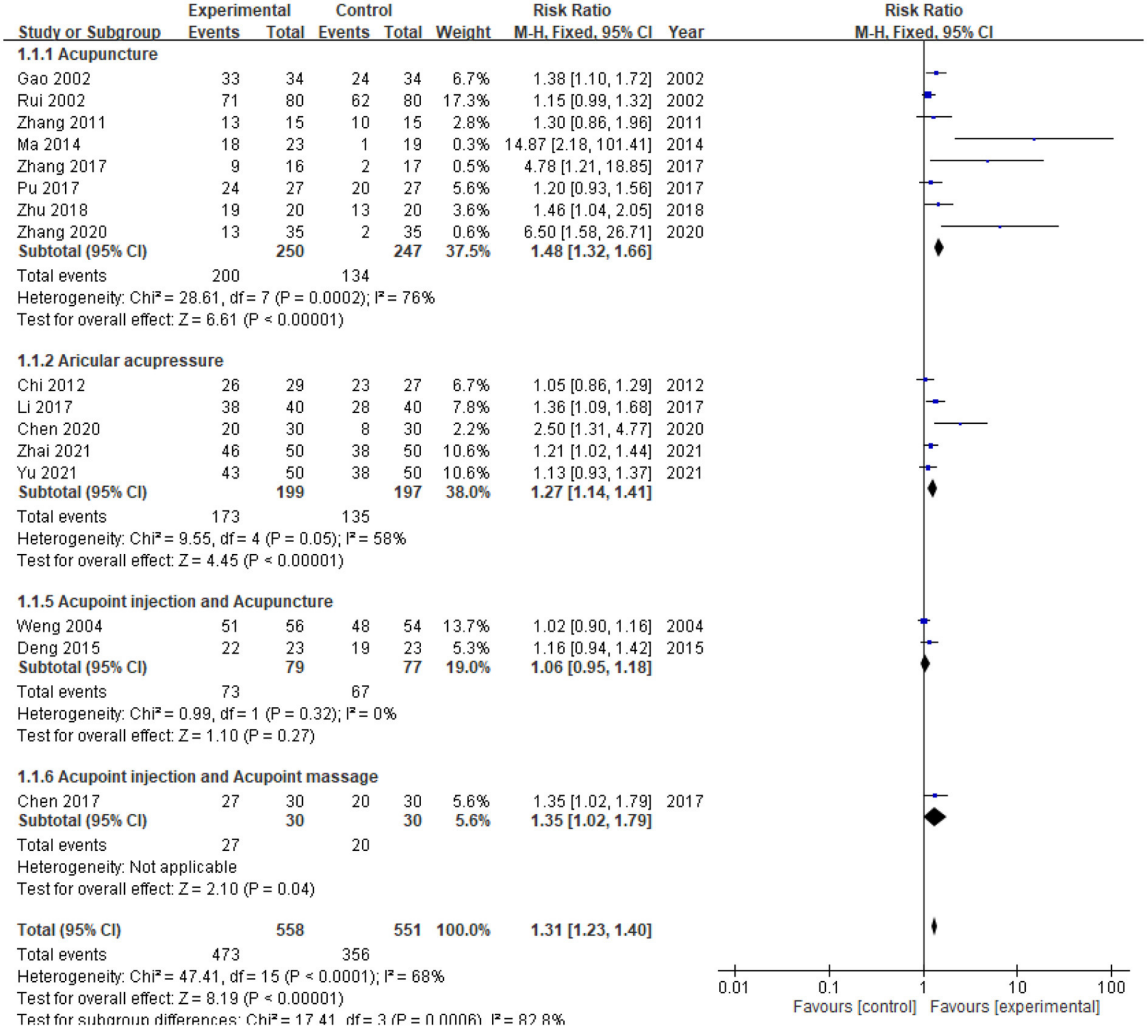
Forest plot comparison of effective rates in patients with uremic pruritus treated with acupuncture or related therapies. CI, confidence interval; df, degrees of freedom; M-H, Mantel-Haenszel.

Nine studies recorded the number of patients that became better after the therapies. For acupuncture, three individual studies showed statistically significantly higher efficacy in the treatment vs. the control group [risk ratio (RR) 4.78–14.87; Figure 4]. Overall results for acupuncture (RR 1.48; 95% CI: 1.32, 1.66), AA (RR 1.27; 95% CI: 1.14, 1.41), and AI+AM (RR 1.35; 95% CI: 1.02, 1.79) all showed increased efficacy relative to control; among these complementary therapies, improvements were statistically significant for acupuncture (*p* < 0.00001), AA (*p* < 0.0001) and AI+AM (*p* = 0.04). Although AI + acupuncture showed no significant improvement in efficacy relative to control, the complementary therapies evaluated demonstrated significantly improved overall efficacy vs. control (RR 1.31; 95% CI: 1.23, 1.40; *p* < 0.00001).

#### Laboratory parameters

**Figure 5:**
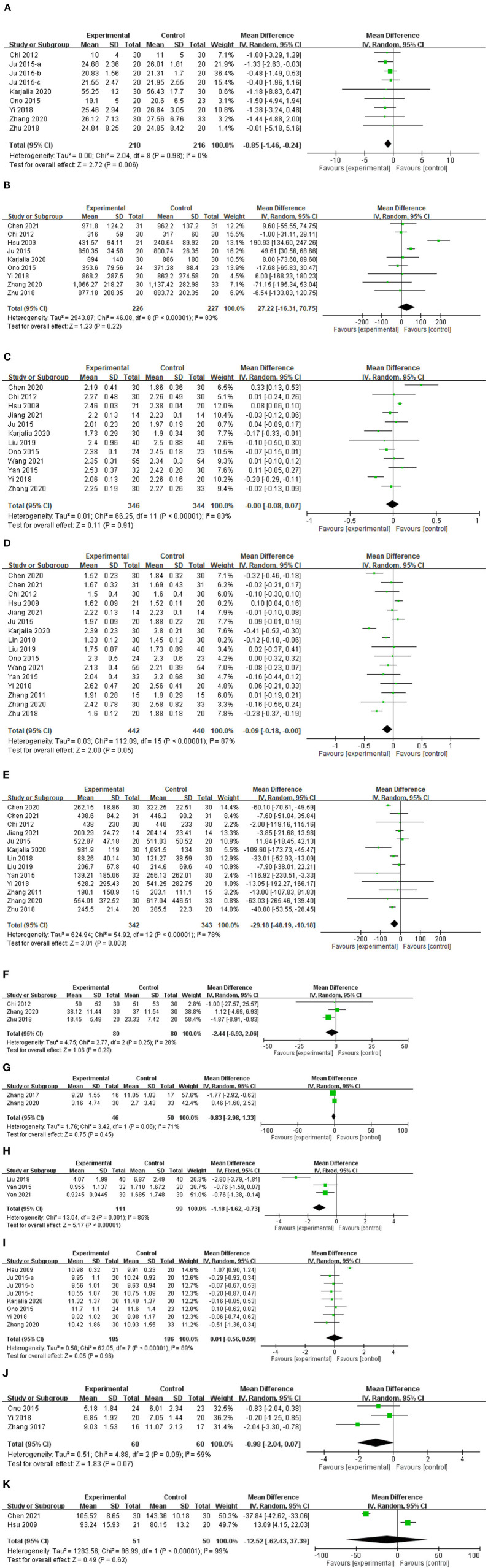
Forest plot comparison of serum levels of **(A)** blood urea nitrogen, **(B)** creatinine, **(C)** calcium, **(D)** phosphate, **(E)** parathyroid hormone, **(F)** β2-microglobulin, **(G)** C-reactive protein, **(H)** histamine, **(I)** hemoglobin, **(J)** white blood cells, and **(K)** alkaline phosphatase in patients with uremic pruritus treated with acupuncture or other related complementary therapies. CI, confidence interval; IV, independent variable; SD, standard deviation.

The table of serum data from the collected studies was attached in the Supplementary material. Statistically significant overall improvement vs. control were noted, and with mild to high heterogeneity, for the complementary therapies evaluated, regarding BUN (MD −0.85; 95% CI: −1.46, −0.24; *p* = 0.006; Figure 5), PTH (MD −29.18; 95% CI: −48.19, −10.18; *p* = 0.003), and histamine levels (MD −1.18; 95% CI: −1.62, −0.73; *p* < 0.0001). Changes in serum creatinine, phosphate, calcium, β2-microglobulin, C-reactive protein, hemoglobin, white blood cell count, and alkaline phosphatase were not statistically significant (p>0.05 for each; moderate to high heterogeneity).

## Discussion

In our review, acupoint acupuncture, AA, AI, AM, AIR, TEAS, and AI+AM statistically significantly improved mean VAS scores for pruritus in patients with UP (9, 12, 14, 27, 29, 34, 40, 42–44, 50, 53). Decreased mean PMS scores for treatment vs. control were noted in three studies: one study about acupuncture, and two about AA (5, 39, 44). Sixteen studies recorded effective rates for acupuncture, AA, acupuncture + AI, and AI+AM: results revealed significantly greater efficacy than control for acupuncture (RR 1.48; 95% CI: 1.32, 1.66), AA (RR 1.32; 95% CI: 1.00, 1.46), and AI+AM (RR 1.35; 95% CI: 1.02, 1.79) (25, 29, 30, 33, 34, 36–38, 41, 46, 48, 51, 53). In large populations, especially, small changes may be statistically significant, but can be irrelevant clinically. The minimal clinically important difference (MCID) is defined as the smallest change in any scale scoring that can be noticed by the patient (57). Several methods exist for determining MCID; however, currently no universal rule is established. Reich et al. suggested that the MCID for clinical improvement in itch, as rated on the VAS and NRS, ranks between a decrease of 2–3 points (57). Claudia et al. found MCID of 192 patients that the very severe baseline pruritus (>9) had to be reduced by at least 4.56 points, and the severe pruritus (7 to <9) by at least 3.65 (58). In our article, improvement of VAS is modest to minor, although statistically significant after pooling to meta-analyze the expanded study population.

Traditionally, acupuncture has been reported as a safe and effective treatment for pruritus, and has been used in China for many years (59). Yan et al. reported that acupuncture significantly reduced pruritus scores in hemodialysis patients with UP (60). Generally, the most frequently used method was manual acupuncture, although TEAS and auricular acupuncture have also been used for UP (14).

In our acupuncture group, the most common acupoint choices were SP10 (eight studies), LI11 (seven studies), SP6, and ST36 (each of these acupoints used in six studies) (5, 22, 25–34, 37, 38). EA at LI11 and SP10 could improve pruritus due to reducing the expression of TLR2, TLR4, MyD88 and NF-κB which increased in the morphine-induced pruritus model mice (61). Cold stimulation (20°C) at LI11 in compound 40/80-induced mice showed decreased c-fos expression in the dorsal horn at C2-C7 and decreased scratching bouts (62). The treatment frequency was about two to three times per week and decreased VAS, PMS, and DRKS scores confirmed the efficacy of the acupuncture. Overall, VAS scores showed significant improvements vs. control for acupuncture, AA, AI, AM, AIR, ATENS, and AI+AM (9, 12, 14, 27, 29, 34, 40, 42–44, 50, 53). Three studies revealed a significant decline in PMS scores, but with significant heterogeneity among the studies (*I*^2^ 99%; *p* < 0.00001) (5, 39, 44). Ma et al., Zhang et al., and Chang et al. all reported improved effective rates after acupuncture therapies compared with control (25, 31, 33). Regarding complications, Che et al. used acupuncture at SP6, SP10, ST36, and LI11 in 40 patients and could report that 2 patients (one in acupuncture group, another in control group) complained about elbow soreness and 3 patients in the control group complained about minimal bleeding induced by acupuncture (5). Phan et al. performed 216 times (12× in 18 patients) in patients undergoing hemodialysis who received heparin. Bleeding was observed in 13 patients after the needle was removed from the spot, and the bleeding was mild and could be controlled with the use of cotton and the application of pressure. No serious cases of bleeding occurred. Hematoma, which occurred after bleeding, was observed in four (1.85%) patients. However, the hematoma disappeared without any therapy within 3–10 days (28).

Auricular acupoints affect the functioning of the visceral organs and meridians, skeleton, and limbs, and AA proved to be a useful treatment for UP in patients with end-stage renal disease (60). Indeed, in patients undergoing hemodialysis, AA had a beneficial effect on conditions such as sleep disorders, depression, pruritus, and xerostomia (63). Karjalian et al. reported improved pruritus in a study that evaluated AM as a treatment for UP (7), and PMS scores improved after AA in studies conducted by Mai et al. and He et al. (39, 44). For AA, the treatment frequency in studies in our review was about three to eight times per day, and the meridians used included lung, kidney, heart, stomach, endocrine, subcortical, supracortical, adrenal, and TF4. One study used auricular acupoint scraping therapy for UP patients (35). Scraping stimulation makes subcutaneous capillaries expand or break, creating skin eruptions with flush or purple-red skin, miliary and papuloid spots, patchy and stripy plaques, and local hot sensation or mild pain. Skin eruptions can improve blood circulation, promote cell metabolism, and strengthen immunity to cure diseases and promote recovery (64). Overall, AA produced significant relief of symptoms, and significantly improved effective rates, relative to control (39, 44).

AI uses a syringe needle instead of an acupuncture needle at acupoints (50). AI achieved the same or higher plasma concentrations than the intravenous injection of carbamyl β -methylcholine chloride injection at ST36 and femoral vein (65). There was a significantly increased phylloquinone plasma concentration found in a study of AI at SP6 as the trigger point (66). Several clinical studies have shown that AI has definite advantages and reliable curative effects for the treatment of pruritis in patients with CKD (51–53, 67).

AM stimulates the meridian points by pressure applied with the fingertips, palms, small beads, or special devices (68). The pressure on acupoints could promote blood circulation and neurotransmitter secretion (7). Chen et al. combined AI with AM for patients with UP and reported improved efficacy relative to a control group (53).

Thermal (including infrared and far infrared) therapy has been used to treat pain, depression, dysmenorrhea, and coronary vascular endothelial dysfunction (49). Far infrared radiation has a wavelength of 4–1,000 μm and can penetrate the subcutaneous tissues; as such, it may improve blood flow and endothelial and nervous-system function (49). Infrared rays can provide local warmth around acupoints and can have a similar effect to acupuncture; such rays may improve skin blood flow and the functioning of the cutaneous nervous system (69). Yi et al. found that AIR is effective in the treatment of UP, as evident from improvements in VAS and sleep quality scores (9).

Umbilical AST may increase immune function and amplify the effect of Traditional Chinese Medicine (TCM) (56). Indeed, for medications applied at the umbilical acupoint, bioavailability may be increased up to 6-fold (56). Jiu et al. used several Chinese medications with antioxidant and antiallergic properties and reported significantly improved VAS scores after AST (*p* < 0.01) (56).

Akca et al. assessed acupressure and TEAS applied at acupoint LI11 (14); TEAS provides a faint electrical current and causes a small amount of pressure at the acupoint. The researchers reported a significant decrease in pruritus severity, as evident from a significant improvement in the mean VAS score (*p* = 0.0003) (14).

Patients with UP have higher serum levels of iPTH, hemoglobin, BUN, and high-sensitivity C-reactive protein rather than patients without UP (70). In our results, there were significant decreases in serum BUN, phosphate, PTH, and histamine levels in patients with UP compared with controls. Increased calcium and phosphate levels increase the stimulation of peripheral nerves in the skin; indeed, Sunita et al. found that calcium phosphate-induced pruritus was mediated by interleukin-6, Bruton's tyrosine kinase, and extracellular signal-regulated kinase signaling in a murine model (71). PTH has also been associated with mast cell activation, leading to histamine release and pruritus (72). In an experimental study, Chinese herbal medicine (CHM) and acupoint thread implantation reduced serum PTH concentration in rats with CKD (73). Stockenhuber et al. observed increased histamine levels in patients with pruritus and CKD (74), and in another study, antihistamine therapy significantly improved the mean VAS score, indicating improved control of UP (75). Importantly, increased levels of calcium, phosphate, PTH, and histamine might cause a pruritic sensation in patients with UP.

A previous meta-analysis collected data from six studies of acupuncture in UP and showed, albeit with insufficient evidence, that acupuncture and acupressure were effective in UP; the researchers included articles in English, and only three trials recorded VAS scores (68). Importantly, our analysis collected data from a total of 36 studies, without language limits, and included ten different acupuncture techniques, several pruritus scores, and overall effective rates. However, the studies included in our analysis had considerable heterogeneity because of various clinical factors: the durations of each acupuncture method varied; some inter-study discrepancies in control groups existed, and overall effective rates were measured using different pruritus scores.

There were some limitations to our analysis: first, sample sizes of the included RCTs were small; second, all the selected articles lacked long-term follow-up; and third, the therapeutic mechanisms of effect for the different acupuncture techniques in UP were unclear. Nonetheless, our analysis is one of the first to endorse the therapeutic benefit of acupuncture and related techniques in the treatment of UP in patients with CKD.

## Conclusion

In summary, our meta-analysis found that acupuncture and related techniques have clinical benefits, as evident from various pruritus scores, in patients with UP. Further investigations in larger study populations and well-designed studies focusing on the dosage, frequency, and long-term effects are now warranted.

## Data availability statement

The original contributions presented in the study are included in the article/Supplementary material, further inquiries can be directed to the corresponding author/s.

## Author contributions

Po-HL designed the study and critically revised the manuscript. Po-HL, C-HC, H-EC, and I-HL contributed to the literature search, data extraction, quality assessment, and writing the first draft of the article. Pi-HL and C-HC performed statistical analysis and interpreted the results. All authors contributed to this article. All authors have read and agreed to the published version of the manuscript.

## Funding

This work was supported by grants from the Buddhist Tzu Chi Medical Foundation, Taiwan (TCMF-CM1-111-03 and TCMF-P 111-16) and Taipei Tzu Chi Hospital (TCRD-TPE-111-45).

## Conflict of interest

The authors declare that the research was conducted in the absence of any commercial or financial relationships that could be construed as a potential conflict of interest.

## Publisher's note

All claims expressed in this article are solely those of the authors and do not necessarily represent those of their affiliated organizations, or those of the publisher, the editors and the reviewers. Any product that may be evaluated in this article, or claim that may be made by its manufacturer, is not guaranteed or endorsed by the publisher.
