# Supplementary material for: Efficacy of acupoint stimulation as a treatment for uremic pruritus: A systematic review and meta-analysis
Source: Front Med (Lausanne). 2022 Dec 1;9:1036072. doi: 10.3389/fmed.2022.1036072 (PMC9751623; doi:10.3389/fmed.2022.1036072)
Supplement: Supplementary file 1 [file Data_Sheet_1.zip › Supplementary Table 1.docx]

Supplementary Material

| Supplementary Table 1. Serum data from the selected studies^a^ | | | | | | | | | | | | | | |
| --- | --- | --- | --- | --- | --- | --- | --- | --- | --- | --- | --- | --- | --- | --- |
| Study (year) | Alb (g/L) | Calcium  (mmol/L) | Phosphorus  (mmol/L) | Creatinine  (μmol/L) | BUN  (mmol/L) | PTH  (pg/ml) | Na  (mmol/L) | K  (mmol/L) | Urea  (mg/dl) | β2-MG  (pmol/L) | Hb  (g/dL) | WBC  (x10^9^/L) | Plt  (x10^9^/L) |  |
| **Acupuncture** | | | | | | | | | | | | | |  |
| Ardinata et al.(54)*  (2021) | NA | NA | NA | C:  1400.26(370.40) | NA | NA | NA | NA | NA | NA | C:  9.52(1.38) | NA | NA |  |
| Fan et al. (74)(2021) | NA | (mg/dL)  T: 9.60(0.70) → 9.10(1.20)  C: 8.50(1.10) → 7.90(0.90) | (mg/dL)  T: 4.10(1.30) → 4.00(1.40)  C: 4.80(0.80) → 4.60(0.90) | (mg/dL)  T: 11.50(2.60) → 12.50(1.80)  C: 10.60(3.10) → 11.10(2.90) | (mg/dL)  T: 84.60(19.90) → 89.60(18.97)  C: 91.20(10.50) → 89.20(11.60) | NA | NA | NA | NA | NA | NA | NA | NA |  |
| Jiang et al. (75) (2021) | NA | T1: 2.27(0.19) → 2.23(0.10)  T2: 2.28(0.19) → 2.22(0.13)  C: 2.31(0.18) → 2.15(0.14) | T1: 1.84(0.09) → 1.68(0.15)  T2: 1.77(0.11) → 1.51(0.19)  C: 1.74(0.24) → 1.83(0.22) | NA | NA | T1: 325.67(58.92) → 204.14(23.41)  T2: 301.12(42.83) → 200.29(24.72)  C: 322.69(71.02) → 346.57(42.71) | NA | NA | NA | NA | NA | NA | NA |  |
| Zhang et al.(42)  (2020) | T: 41.67(3.72) → 42.90(2.91)  C: 40.47(3.25) → 39.18(2.78) | T: 2.26(0.31) → 2.25(0.19)  C: 2.23(0.37) → 2.27 (0.26) | T: 2.66(0.65) →2.42(0.78)  C: 2.63(0.85) → 2.58 (0.82) | T: 1117.03(274.18) → 1066.27(218.27)  C: 1127.64(288.86) → 1137.42 (282.98) | T: 27.97(7.11) → 26.12(7.13)  C: 28.01(5.48) → 27.56(6.76) | T: 619.55(268.28-992.51) → 540.34(34.10~84.85)  C: 456.88(321.56~766.00) → 653.50(310.72-886.90) | NA | NA | NA | T: 33.00(7.73) → 38.12(11.44)  C: 31.52(9.93) → 37.00(11.54) | T: 9.77(1.52) → 10.42(1.86)  C: 10.54(1.60) → 10.93(1.55) | NA | NA |  |
| Liu et al.(55) (2019) | NA | T: 1.98(0.67) → 2.40(0.96)  C: 1.95(0.64) → 2.50 (0.88) | T: 2.64(0.95) → 1.75(0.87)  C: 2.65(0.92) → 1.73 (0.89) | NA | NA | T: 335.86(87.8) → 206.70(67.80)  C: 354.85(89.90) → 214.60 (69.60) | NA | NA | NA | NA | NA | NA | NA |  |
| Nahidi et al.(34)  (2018) | NA | NA | NA | NA | NA | NA | NA | NA | NA | NA | NA | NA | NA |  |
| Phan et al.(56)  (2018) | NA | NA | NA | NA | NA | NA | NA | NA | NA | NA | NA | NA | NA |  |
| Chu et al.(35) (2018) | NA | NA | T: 2.28(0.22) → 1.60(0.12)  C: 2.32(0.20) → 1.88 (0.18) | T: 875.45(226.53) → 877.18(208.35)  C: 880.46(210.26) → 883.72 (202.35) | T: 25.28(8.65) → 24.84(8.25)  C: 25.42(8.17) → 24.85(8.42) | T: 324.30(23.50) → 245.50(21.40)  C: 326.50(24.20) → 285.50 (22.30) | NA | NA | NA | T: 32.25(11.20) → 18.45(5.48)  C: 32.75(10.72) → 23.32(7.42) | NA | NA | NA |  |
| Pu et al.(43) (2017) | NA | NA | NA | NA | NA | NA | NA | NA | NA | NA | NA | NA | NA |  |
| Chang et al.(25)  (2017) | NA | NA | NA | NA | NA | NA | NA | NA | NA | NA | NA | T_A_:12.18(1.83) → 9.03(1.53)  T_C_:12.17(1.75) → 5.38(1.69)  C:12.08(1.83) → 11.07(2.12) | NA |  |
| Ono et al.(57)  (2015) | T: 24.83(1.38) → 24.14(1.38)  C: 25.52(1.38) → 24.83(0.69) | T:  2.38(0.15) → 2.38(0.10)  C: 2.43(0.15) → 2.45 (0.18) | T: 2.40(0.50) → 2.30(0.50)  C: 2.60(0.60) → 2.30 (0.60) | T: 362.44(97.24) → 353.60(79.56)  C: 397.80(88.40) → 371.28 (88.40) | T: 22.70(5.90) → 19.10(5.00)  C: 21.44(8.20) → 20.60(6.50) | NA | NA | NA | NA | NA | T: 11.70(1.10) → 11.70(1.00)  C: 12.10(1.40) → 11.60(1.40) | T: 36.67(4.07) → 37.11(6.52)  C: 35.26(3.73) → 34.06(5.57) | NA |  |
| Ma et al.(44) (2014) | NA | NA | NA | NA | NA | NA | NA | NA | NA | NA | NA | NA | NA |  |
| Chang et al.(36)  (2011) | NA | NA | A+HDF: 2.60(0.98) → 1.91(0.28)  HDF: 2.57(0.97) → 1.90(0.29)  C: 2.58(0.95) → 2.31(0.34) | NA | NA | A+HDF: 300.40(194.90) → 190.10(150.90)  HDF: 299.10(197.50) → 203.10(111.10)  C: 303.80(187.20) → 286.60(163.00) | NA | NA | NA | NA | NA | NA | NA |  |
| Che et al.(40)*  (2005) | NA | T:  2.40(0.18)  C:  2.13 (0.28) | T:  1.32(0.42)  C:  1.55 (0.26) | NA | NA | T:  217.30(123.00)  C:  223.50(162.00) | NA | NA | T:  84.60(19.90)  C:  91.20(10.50) | NA | NA | NA | NA |  |
| Ruei et al. (45) (2002) | NA | NA | NA | NA | NA | NA | NA | NA | NA | NA | NA | NA | NA |  |
| Kao et al. (46) (2002) | NA | NA | NA | NA | NA | NA | NA | NA | NA | NA | NA | NA | NA |  |
| **Auricular acupressure** | | | | | | | | | | | | | |  |
| Mai et al.(41) (2021) | NA | NA | NA | NA | NA | NA | NA | NA | NA | NA | NA | NA | NA |  |
| Yan et al.(37)* (2021) | NA | T: 1.95(0.14)  C: 1.92(0.14) | T: 1.84(0.22)  C: 1.90(0.16) | NA | NA | T: 287.00(63.85)  C: 259.50(92.84) | NA | NA | NA | NA | NA | NA | NA |  |
| Yu et al. (27) (2021) | NA | NA | NA | NA | NA | NA | NA | NA | NA | NA | NA | NA | NA |  |
| Zhai et al.(47)  2021 | NA | NA | NA | NA | NA | NA | NA | NA | NA | NA | NA | NA | NA |  |
| Chen et al.(24)*  (2020) | NA | AA+ND: 2.19(0.41)  ND: 1.86(0.36)  C: 1.74(0.32) | AA+ND: 1.52(0.23)  ND: 1.84(0.32)  C: 1.98(0.36) | NA | NA | AA+ND: 262.15(18.86  ND: 322.25(22.51)  C:  362.25(25.45) | NA | NA | NA | NA | NA | NA | NA |  |
| Yan et al.(38)* (2020) | NA | T: 2.46(0.32)  C: 2.37(0.34) | T: 2.14(0.48)  C: 2.28(0.65) | NA | NA | T: 283.00(341.20)  C: 353.50(422.30) | NA | NA | NA | NA | NA | NA | NA |  |
| Ding et al.(23)*  (2019) | NA | T: 1.97(0.15) → 2.10(0.14)  C: 1.95(0.19) → 2.61 (0.59) | T: 1.86(0.26) → 1.73(0.19)  C: 1.75(0.42) → 1.72 | NA | NA | T: 299.52(71.68) → 210.50(48.50)  C: 269.25(109.21) → 239.06 (91.98) | NA | NA | NA | NA | T:  9.82(1.27)  C:  9.90(0.99) | NA | NA |  |
| He et al.(39)* (2018) | T:  40.75(2.87)  C:  41.03(3.24) | T:  2.25(0.21)  C:  2.19(0.23) | T:  2.03(0.73)  C:  2.18(0.71) | T:  974.00(282.72)  C:  1092.46(316.41) | NA | T:  373.68(426.46)  C:  512.91(506.85) | NA | NA | T:  57.72(199.95)  C:  25.02(6.74) | NA | NA | NA | NA |  |
| Lin et al.(26) (2018) | NA | NA | Fumigation:  2.13(0.11) → 1.32(0.13)  AA: 2.13(0.12) → 1.33(0.12)  Fumigation+AA: 2.14(0.10) → 1.32(0.12)  C: 2.12(0.10) → 1.45(0.12) | NA | NA | Fumigation:  179.81(26.33) → 87.32(40.25)  AA: 178.31(26.06) → 88.26(40.14)  Fumigation+AA: 177.13(26.21) → 89.15(40.37)  C: 176.81(26.33) → 121.27(38.59) | NA | NA | NA | NA | NA | NA | NA |  |
| Li et al.(48)  (2017) | NA | NA | NA | NA | NA | NA | NA | NA | NA | NA | NA | NA | NA |  |
| Tao et al.(76) (2016) | NA | NA | NA | NA | NA | NA | NA | NA | NA | NA | NA | NA | NA |  |
| Yan et al.(13) (2015)  %: median(Q1-Q3) | NA | T: 2.48(0.35) →2.53(0.37)  C: 2.39(0.39) → 2.42 (0.28) | T: 2.13(0.51) →2.04(0.40)  C: 2.25(0.66) → 2.20 (0.68) | NA | NA | (pg/L)  †T: 189.0(92.90-339.0) → 119.5(29.83-268.3)  †C: 239.0(98.90-435.5)→ 234.0(98.90-435.5) | NA | NA | NA | NA | NA | NA | NA |  |
| Shr et al.(49) (2012) | NA | T:  1.93(0.35) → 2.27(0.48)  C1(HD+HP): 1.92(0.37) → 2.26(0.49)  C2(HD): 1.94(0.36) → 1.97(0.18) | T:  2.80(0.50) → 1.50(0.40)  C1(HD+HP): 2.70(0.40) → 1.60(0.40)  C2(HD): 2.80(0.40) → 2.50(0.40) | T:  820(273) → 316(59)  C1(HD+HP): 818(275) → 317(60)  C2(HD): 810(281) → 310(64) | T:  31(5) → 10(4)  C1(HD+HP): 30(6) → 11(5)  C2(HD): 29(6) → 12(3) | (ng/L)  T:  980(273) → 438(230)  C1(HD+HP): 978(275) → 440(233)  C2(HD): 979(270) → 878(268) | NA | NA | NA | T:  76(72) → 50(52)  C1(HD+HP): 75(73) → 51(53)  C2(HD): 73(75) → 72(74) | NA | NA | NA |  |
| **Acupoint far infrared** | | | | | | | | | | | | | |  |
| Hsu et al.(66) (2009) | T:  3.97(0.06) → 3.98(0.08)  C:  3.72(0.08) → 3.78(0.07) | T:  2.50(0.04) → 2.46(0.03)  C:  2.44(0.05) → 2.38(0.04) | T:  1.78(0.12) → 1.62(0.09)  C:  1.63(0.13) → 1.52(0.11) | T:  322.40(74.46) → 431.57(94.11)  C:  154.33(60.27) → 240.64(89.92) | NA | NA | NA | NA | (No unit)  T:  1.39(0.05) → 1.46(0.08)  C:  1.54(0.09) → 1.75(0.10) | NA | T:  11.01(0.36) → 10.98(0.32)  C:  10.22(0.28) → 9.91(0.23) | NA | NA |  |
| **Acupoint injection** | | | | | | | | | | | | | |  |
| Wang et al.(28)  (2021) |  | T:  2.38(0.37)→ 2.35(0.31)  C:  2.34(0.31) → 2.34(0.30) | T:  2.14(0.53)→ 2.13(0.40)  C:  2.17(0.52) → 2.21(0.39) | T:  577.5(166.48) → 493.96(126.46)  C:  536.78(163.12) → 183.59(112.02) | NA | NA | NA | NA | NA | NA | NA | NA | NA |  |
| **Acupoint injection and Acupuncture** | | | | | | | | | | | | | |  |
| Deng et al.(31)  (2017) | NA | NA | NA | NA | NA | NA | NA | NA | NA | NA | NA | NA | NA |  |
| Wang et al.(32)  (2004) | NA | NA | NA | NA | NA | NA | NA | NA | NA | NA | NA | NA | NA |  |
| **Acupoint injection + Acupoint massage** | | | | | | | | | | | | | |  |
| Chen et al.(33)  (2017) | NA | NA | NA | NA | NA | NA | NA | NA | NA | NA | NA | NA | NA |  |
| **Acupoint infrared** | | | | | | | | | | | | | |  |
| Yi et al.(9) (2018) | T:  39.25(4.18) → 40.66(3.45)  C:  40.11(3.94) → 40.55(4.02) | T:  2.35(0.18) → 2.06(0.13)  C:  2.43(0.21) → 2.26(0.16) | T:  2.54(0.55) → 2.62(0.47)  C:  2.47(0.52) → 2.56(0.41) | T:  970.62(240.18) → 868.20(287.50)  C:  890.60(247.63) → 862.20(274.58) | T:  26.44(3.22) → 25.46(2.94)  C:  25.73(2.82)→ 26.84(3.05) | T:  547.60(302.12) → 528.20(295.43)  C:  527.57(277.34) → 541.25(282.75) | NA | NA | NA | NA | T:  9.86(1.28) → 9.92(1.02)  C:  10.06(1.04) → 9.98(1.17) | T:  7.92(2.12) → 6.85(1.92)  C:  7.22(2.12) → 7.05(1.44) | T:  174.60(40.18) → 168.20(37.53)  C:  159.60(47.66) → 162.20(44.51) |  |
| Acupoint massage | | | | | | | | | | | | | |  |
| Chen et al. (29) (2021) | NA | NA | T: 2.16(0.52) → 1.67(0.32)  C: 2.23(0.74) → 1.69(0.43) | T: 963.80(112.60) → 971.80(124.20)  C: 974.50(123.70) → 962.20(137.20) | NA | T: 579.40(100.30) → 438.60(84.20)  C: 583.80(100.30) → 446.20(90.20) | NA | NA | NA | NA | NA | NA | NA |  |
| Karjalian et al.(7) (2020) | NA | T:  1.83(0.32) → 1.73(0.29)  C1(pressure on ineffective point):  0.32(0.19) → 1.90(0.34)  C2(no intervention): 1.90(0.19) → 1.79(0.27) | T:  2.84(0.30) → 2.39(0.23)  C1(pressure on ineffective point):  2.76(0.26) → 2.80(0.21)  C2(no intervention: 2.78(0.18) → 2.81(0.23) | (No unit)  T:  1114.9(200.2) → 981.9(119)  C1(pressure on ineffective point):  1056.1(168.4) → 1091.5(134)  C2(no intervention: 1002.6(126) → 1111.5(206.6) | (No unit)  T:  54.97(12.74) → 55.25(12.00)  C1(pressure on ineffective point):  55.23(19.28) → 56.43(17.70)  C2(no intervention: 59.13(14.00) → 60.83(12.50) | (No unit)  T:  1114.9(200.2) → 981.9(119)  C1(pressure on ineffective point):  1056.1(168.4) → 1091.5(134)  C2(no intervention: 1002.6(126) → 1111.5(206.6) | T:  140.13(3.50) → 138.63(3.70)  C1(pressure on ineffective point):  139.77(4.34) → 138.10(3.54)  C2(no intervention: 137.00(4.03) → 138.60(3.92) | T:  6.10(0.81) → 6.56(1.38)  C1(pressure on ineffective point):  6.16(1.10) → 6.65(1.27)  C2(no intervention: 5.95(0.80) → 6.65(1.79) | NA | NA | T:  11.50(1.42) → 11.32(1.37)  C1(pressure on ineffective point):  11.47(1.42)→ 11.48(1.37)  C2(no intervention: 11.34(1.37) → 11.34(1.37) | NA | NA |  |
| Akca et al.(15)  (2016) | NA | NA | NA | NA | NA | NA | NA | NA | NA | NA | NA | NA | NA |  |
| Jedras et al.(77)*  (2003) | NA | (mg%)  T:  1.43(0.42)  C:  1.22(0.20) | (mg%)  T:  6.47(1.58)  C:  6.08(1.39) | (mg%)  T:  11.31(4.67)  C:  11.66(4.68) | NA | T:  381.00(506.22)  C:  256.50(431.55) | NA | NA | (mg%)  T:  159.47(39.42)  C:  176.47(42.76) | NA | NA | NA | NA |  |
| **Acupoint sticking therapy** | | | | | | | | | | | | | |  |
| Jiu et al.(30) (2015) | NA | T1(HD):  2.29(0.20) → 2.01(0.23)  C1(HD)  2.28(0.18)→ 1.97(0.19)  T2(HD+HPF):  2.09(0.19) → 2.33(0.17)  C2(HD+HPF)  2.14(0.23) → 2.28(0.18)  T3(HD+HF):  2.21(0.22)→ 2.38(0.11)  C3(HD+HF):  2.21(0.13)→ 2.39(0.10) | T1(HD):  2.29(0.11) → 1.97(0.09)  C1(HD)  2.19(0.21) → 1.88(0.22)  T2(HD+HPF):  2.04(0.25) → 1.65(0.37)  C2(HD+HPF)  2.07(0.22) → 1.72(0.36)  T3(HD+HF):  2.37(0.11) → 2.25(0.12)  C3(HD+HF):  2.36(0.11)→ 2.24(0.13) | T1(HD):  907.64(36.91) → 850.35(34.58)  C1(HD)  583.22(26.83) → 800.74(26.35)  T2(HD+HPF):  1016.86(29.48)→ 687.95(20.99)  C2(HD+HPF)  989.30(25.03) → 698.65(20.81)  T3(HD+HF):  856.21(37.31) → 653.11(29.44)  C3(HD+HF):  844.36(36.03) → 678.72(29.44) | T1(HD):  30.49(2.72) → 24.68(2.36)  C1(HD)  30.41(2.01) → 26.01(1.81)  T2(HD+HPF):  29.40(1.27) → 20.83(1.56)  C2(HD+HPF)  29.34(1.26) → 21.31(1.70)  T3(HD+HF):  30.26(2.56) → 21.55(2.47)  C3(HD+HF):  30.69(2.56) → 21.95(2.55) | T1(HD):  576.16(50.51) → 522.87(47.18)  C1(HD)  565.71(50.57) → 511.03(50.52)  T2(HD+HPF):  653.39(52.62) → 546.79(43.94)  C2(HD+HPF)  648.49(52.47) → 548.39(43.74)  T3(HD+HF):  614.07(71.50)→ 227.26(50.82)  C3(HD+HF):  627.99(85.28) → 242.75(54.82) | NA | NA | NA | NA | T1(HD):  9.72(1.05) → 9.95(1.10)  C1(HD)  9.87(0.84) → 10.24(0.92)  T2(HD+HPF):  8.90(1.13) → 9.56(1.01)  C2(HD+HPF)  9.03(1.23) → 9.63(0.94)  T3(HD+HF):  9.62(1.08) → 10.55(1.07)  C3(HD+HF):  9.62(1.10) → 10.75(1.09) | NA | NA |  |
| **Acupoint transcutaneous electrical nerve stimulation** | | | | | | | | | | | | | |  |
| Akca et al.(15) (2016) | NA | NA | NA | NA | NA | NA | NA | NA | NA | NA | NA | NA | NA |  |
| a: a Values expressed as mean ± SD  *: Values are expressed as mean±SD in baseline data  †: Values are expressed as median and 25% percentile - 75% percentile  SD: standard deviation, T: treatment group, C: control group, NA: not applicable, HD: hemodialysis, HDF: hemodiafiltration, HF: hemofiltration, HP: hemoperfusion, AA: auricular acupressure | | | | | | | | | | | | | | |
